# Applications of Chitosan Nanoparticles in Dentistry: A Review

**DOI:** 10.7759/cureus.49934

**Published:** 2023-12-04

**Authors:** Aastha Agrawal, Amit Reche, Sakshi Agrawal, Priyanka Paul

**Affiliations:** 1 Public Health Dentistry, Sharad Pawar Dental College and Hospital, Datta Meghe Institute of Higher Education and Research, Wardha, IND; 2 Public Health Dentistry, Sharad Pawar Dental College And Hospital, Datta Meghe Institute of Higher Education and Research, Wardha, IND

**Keywords:** drug delivery, biomaterials, dentistry, nanoparticles, chitosan

## Abstract

Recently, nanotechnology has garnered significant interest across various fields due to its emerging and diverse applications. Numerous investigators have proposed that chitosan nanoparticles (CSNPs) stand out as some of the most promising nanomaterials for facilitating various activities. Chitosan, a natural biopolymer established through the deacetylation of chitin, has been extensively studied using interdisciplinary approaches for a wide range of applications. Chitosan biomaterials exhibit distinctive attributes, including biodegradability, muco-adhesion, and biocompatibility, as well as a broad spectrum of antibacterial and antifungal properties. Furthermore, chitosan stands as the sole naturally occurring cationic polysaccharide, and it can be chemically tailored into various derivatives, depending on the intended role and utilization. The potential applications of chitosan are vast and intriguing, with many yet to be fully explored and understood. The unique characteristics of chitosan have sparked growing attentiveness in pharmaceutical industries and biomedical areas around the globe. The characteristics of chitosan like its biocompatibility, and anti-inflammatory effects hold the potential to yield promising outcomes in wound healing and periodontal care following tooth extractions. The objective of this study is to provide an overview of potential applications of chitosan in the field of dentistry.

## Introduction and background

Biomaterials are natural, synthetic, or semisynthetic substances that have achieved significant progress over recent years [1]. Biomaterials have been used successfully in clinical areas to reinstitute the structure and supplant or improve the capacity of organs or tissues in the field of dentistry and various surgeries [2]. Oral cavity infectious disorders such as periodontitis, candidiasis, tooth caries, and endodontic infections are caused due to various reasons. As a result, enormous efforts have been made to cure and prevent infectious disorders of the oral cavity and to improve its various functions. One of the favorable natural biomaterials is chitosan which has numerous biomedical applications. A cationic polymer made of N-acetylglucosamine and D-glucosamine, chitosan is a chitin derivative [3]. A variety of chitosan derivatives have recently been created to enhance its antimicrobial, antifungal, and antibacterial properties [4,5]. Chitosan nanoparticles (CS-NPs) have revealed better biological effects such as antioxidant, anticancer, and anti-inflammatory actions. Because of its qualities of biocompatibility, low allergenicity, and biodegradability, chitosan has gained recognition as a useful biopolymer [6].

Chitin is the second most prevalent naturally occurring biopolymer, characterized by its linear structure and high molecular weight, and is frequently recovered from marine shell waste. Chitosan is an FDA-approved copolymer obtained from plant as well as animal sources. The exoskeleton of crustaceans like crabs and shrimp continues to be the principal source of chitin. Additionally, sources of chitin encompass insects, fungi, and specific plants like mushrooms [3,7]. Due to its adaptability in many forms, including scaffolds, capsules, films, powder, bandages, and hydrogels, chitosan has garnered a lot of enthusiasm in the area of biomedicine. Due to the presence of its amino groups, CS is a biopolymer that exhibits a cationic character [6,8]. Although this nanoparticle is extremely dissolvable in acidic solutions with a pH lower than '6.0' because of the amino group, its solubility is not ideal for biological applications. Improving the solubility increases the applications of these nanomaterials. There are practical ways to accomplish this goal, including deacetylation of chitin and chemical variation by attaching hydrophilic biomolecules to amino or hydroxyl groups [7].

One of the important challenges for any dental material is to withstand the difficult circumstances of the oral environment by staying biologically active. These nanoparticles are acquiring popularity in dentistry because of their amazing biological characteristics. One of the most fascinating properties of chitosan is the capacity to develop new biomaterials due to its high bioactivity which can be used for more applications in dentistry. The scientific development in biomaterials have permitted a substantial development in this field which focuses on new biomaterials leading to various clinical area. The aim of this review is to emphasize and elaborate on the role of CSNPs in the diagnosis, prevention, and treatment of oral diseases.

## Review

Rationale 

To the knowledge of the authors, the literature shows a lack of evidence compiling the properties, applications, and limitations of Chitosan-based nanoparticles in dentistry. Hence, to bridge this knowledge gap this review article has been made. Gaining knowledge regarding this would enhance its use in almost every branch of dentistry including periodontics, endodontics, implantology, pulp regeneration therapy, and many more. These materials can be used for patients who are undergoing dental treatments like restorations, caries prevention, regeneration procedures, and any person who is willing to undergo modified oral hygiene procedures. The added properties of this material will ultimately contribute to better patient compliance and treatment outcomes.

Methodology

**Figure 1:**
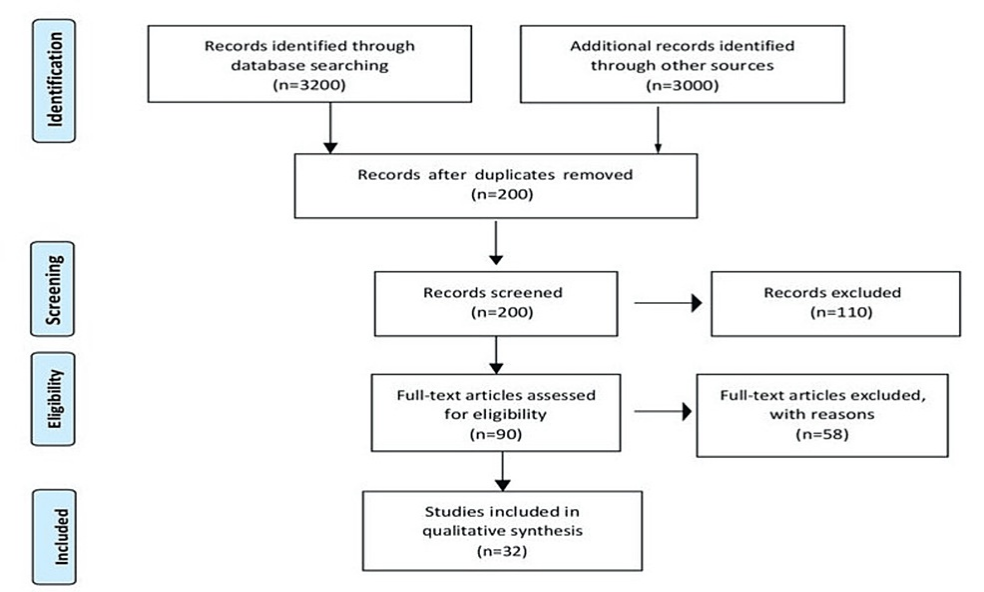
PRISMA flow diagram for search strategy. Adopted from PRISMA. PRISMA, Preferred Reporting Items for Systematic Reviews and Meta-Analyses.

We gathered literature through PubMed, Google Scholar, Scopus, and Web of Science in September 2023. Keywords such as “chitosan, nanoparticles, dentistry, biomaterials, endodontic.” were used. The search was updated in June 2023. The study selection procedure included screening titles and abstracts, followed by full-text evaluation. This review included relevant books, articles, and reviews. The studies in languages other than English were excluded because of limitations of resources and full-text articles were unavailable to the reviewer. The study selection procedures included screening titles and abstracts followed by full-text evaluation. The final group of included studies offers a thorough analysis that is recently available on chitosan applications in dentistry and the results were combined to draw meaningful outcomes. Figure 1 describes the selection process of articles used in our study.

Chitosan

In 1859, Rouget stumbled upon the discovery of chitosan by subjecting chitin to a heated solution of potassium hydroxide. Gilson provided the existence of glucosamine in chitin and the name chitosan given by Hopper-Seyler in 1894 [9]. The word “chitosan” typically refers to cationic copolymers made up of “ß (1-4) bonded 2-amino-2-deoxy-D-glucose (60%-100%)” and “2-acetamino-2-deoxy-D-glucoside (0%-50%)” [10,11]. The primary product of chitin deacetylation is chitosan, which has a mean degree of deacetylation with a free NH 2 group percentage greater than 60% [12,13]. Chitosan strengthens the development of osteoblasts in bone tissue, which speeds up bone formation. It also has the potential to regenerate connective tissue. In animal models, chitosan is easily modified for the production of implantable biomaterials [14]. It has been investigated as an antibacterial agent against a diverse range of target microorganisms, such as algae, bacteria, yeasts, and fungi, in both in vitro and in vivo trials [15]. It exhibits potent antimicrobial activity in vitro against numerous pathogenic oral cavities like Actinobacillus actinomycetemcomitans, Porphyromonas gingivalis, and Streptococcus mutans which are responsible for plaque production and periodontal disease as well as high effectiveness in decreasing dental plaque [16].

Properties

Bioactivity

Lately, Chitosan seems to be showing great promise in the development of new nanomaterials. Chitosan and calcium-phosphate mineral associations sustain an increased level of bioactivity [17]. The positively charged amino groups, NH3+, may communicate with the electrostatically negatively charged surface of bacterial cells to destroy the cell wall. This can result in leakage of the content of cells. Chitosan has shown antibacterial activity against oral microorganisms [18].

Wound Healing

During dental treatments, heavy bleeding can occur. So, dentists need to be ready with a hemostatic agent. A conducted study focused on assessing the effectiveness of this nanoparticle on wound healing and hemostasis, and the findings yielded positive results. The study demonstrated that chitosan was effective in halting bleeding after dental extraction [19]. Several in vitro investigations showed that the chitosan's characteristics are beneficial in each of them. Chitosan exhibits immunomodulatory characteristics and prompts the release of IL-1 from macrophages. This, in turn, augments fibroblast proliferation and collagen production. Following chitosan application, wounds showed a rise in osteopontin and collagen as well as a significant infiltration of polymorphonuclear (PMN) leukocytes. Since chitosan has a higher degree of deacetylation than chitin, it appears to have more active fibroblasts and is more resistant to bursting wounds [15].

Bone Repair

There are several major illnesses that harm the bones and are very difficult to treat. Examples of these include the removal of tumors and osteolysis. It is crucial to advance bone repair techniques and look for new materials as invasive surgical procedures become more prevalent in specialties like orthopedics and dentistry. The use of these materials should result in shorter treatment times, smaller scars, less post-operative pain, and quicker patient recovery [20,21].

Chitosan's biodegradability and biocompatibility allow for this biomaterial used in the hard tissue repair process. Its mechanism runs on the concept of providing a transient scaffold, allowing time to gradually dissolve for the implant and be substituted by natural bone tissue. The characteristics, encompassing elements like hydrogen bonds, polymer chains, cross-linkages, and interactions of NH2+ with negatively charged tissues in the human body, are noticeably affected by its chemical configurations. This imparts to chitosan a notable level of stability, providing a framework for the generation of new bone cells Many studies have showcased the potent impact of chitosan's capabilities on the process of bone repair and regeneration. Chitosan in sponge form has been proven in some studies to activate osteoblasts, which may promote osteogenesis [22].

A study was conducted to investigate how chitosan affects the healing of dental sockets after tooth extraction, particularly in terms of its suitability as a biomaterial for bone regeneration. The results after 10 weeks were intriguing. The middle and apical portions of the treated sockets had significantly greater bone density. Regenerated bone can have a mandibular bone density of up to 98.2%. The epical and central parts of the bone density in each patient rose by 29.3% and 10,8%, respectively, compared to normal bone density. The outcomes show that bone tissue regeneration will occur more quickly in chitosan-filled dental sockets than in untreated dental sockets [23,24].

Anti-inflammatory

The body's innate response to detrimental chemical, physical, or biological stimuli is known as inflammation. We encounter specific periodontal disorders that produce this condition frequently in dentistry. Studies have revealed that Chitosan and its derivatives exhibit diverse effects on this procedure. N-acetyl-D-glucosamine, which encourages the presence of inflammatory cells like macrophages, fibroblasts, and PMN neutrophils, is associated with it [15]. According to studies examining the impact of CSNPs on pathogens in gingival and periodontal fibroblasts, chitosan may prove to be a beneficial substance for treating inflammation in the periodontium. But whether similar success will be found in in vivo studies remains to be seen. Therefore, additional research is needed in this area [24,25].

Applications

Chitosan-based materials have undergone extensive research for various dental applications.

Drug Delivery

Chitosan-based composites (CBCs) offer the potential for developing long-lasting local drug delivery systems possessing the obligatory mechanical characteristics, appropriate contact duration, and sustained release profile, all while retaining a strong affinity with the oral mucosa. CBC increases bioavailability and is used to treat a variety of oral diseases. CHS administration orally is safe. CSNPs and resorbable films can facilitate the transfer of antibiotics like metronidazole, chlorhexidine, and nystatin directly to periodontal tissues. This targeted approach aids in combating fungal infections and oral mucositis effectively in situ. By suppressing certain dental plaque bacterium A. actinomycetemcomitans, S. mutants, and P. gingivalis in vitro, CHS has demonstrated efficient plaque management in dentistry. So far, obtaining a thorough grasp of the antibacterial characteristics of CHS-based materials has been a challenging endeavor [8,14].

Guided Tissue Regeneration

The conception of guided tissue regeneration (GTR) has sparked growing interest in the advancement of regenerative periodontal management techniques. The core principle of GTR involves using an appropriate membrane (either resorbable or non-resorbable) to isolate the periodontal defect. This membrane serves as a physical barrier, preventing gingival tissue from infiltrating into the bone damage. This, in turn, fosters bone regeneration while discouraging the proliferation of fibrous tissue simultaneously. Chitosan has gained recognition as a favorable nanomaterial for guided tissue regeneration, thanks to its alignment with the previously mentioned characteristics [14].

Modifications of Dentifrices

The economically accessible fluoride-free chitosan-based dentifrice, known as Chitodent (B&F), demonstrated a notable depletion in tissue loss.

Enamel Repair

It is difficult to repair or regenerate tooth enamel because it is the toughest and least vascular tissue in the human body. Several research studies have inquired and observed for efficiently delivering organic amelogenin to locations of enamel defects. Currently, a hydrogel made of chitosan was developed with the purpose of delivering amelogenin and revitalizing aligned crystal structure [14].

Dentin Bonding and Adhesion

Researchers are focusing their interest on the interface between dentin and the restoration material, as well as the enduring strength of the bond between them. The elimination of the smear layer and the procedure sensitivity of acid etching are two current problems with dentine replacement materials. A precarious hybrid layer susceptible to nano leakage, results from the insufficient elimination of the smear layer, which frequently leads to deficient resin monomer penetration. Hence, there is a special need for dedicated research in the realm of dentine replacement chitosan-based materials, and more broadly, in the field of bioadhesive polymers. To enhance shear bond strength and create robust dentine bonding systems, researchers investigated antioxidant chitosan hydrogels infused with propolis, nystatin, and β-carotene.

Modification of Dental Restorative Materials

Glass ionomers, which combine poly (acrylic) acid liquid and fluoro aluminosilicate glass powder, create a chemical bond with the calcified tooth tissues. For applications like the cementation of prostheses and restorations, Glass ionomer cements (GICs) are frequently utilized. These systems have been subjected to numerous enhancements, including modifications with resin or nano-additives.

Coating of Dental Implants

Several research have shown that coating dental implants with chitosan has positive benefits. By changing biological, mechanical, and morphological surface qualities, the chitosan coating may have an impact on the surface and bone interaction.

Stem-Based Regenerative Therapeutic

The speedy advancement in the area of stem cell research has facilitated the usage of CSNP as a medium for the repair of stem cells. Utilizing the regenerative capacity of mobilized dental pulp stem cells, researchers have studied the regeneration of the dentine-pulp complex. Another projected objective of current research clusters is the regeneration of the complete tooth. Utilizing various stem cells, tooth engineering has been developed to create dental structures in vivo. Moreover, mesenchymal stem/stromal cells were previously implemented in clinical settings for alveolar bone augmentation, showcasing the effectiveness of stem cell technology in regenerative therapies. 

Endodontic Agents

Endodontic therapies' main objective is to lessen or eliminate the microbial burden in the root canal system. In this context, a number of intracanal medications and root canal irrigants are created as well as tested against both mono- and multi-species biofilms. The antibiofilm action of CSNPs against multispecies infections involving S. mutans, C. albicans, and Enterococcus faecalis is notable. Incorporating CSNPs into calcium hydroxide pastes has been shown to offer benefits as an interappointment medication, as well as in endodontic sealers for filling root canals [26,27].

Anti-fungal Agents

Numerous research studies have examined how chitosan coating on medical devices affects the probability of contamination. Because of chitosan's significant antifungal properties, incorporating its modified forms into substances like tissue conditioners or denture adhesives is anticipated to be an effective method for treating similar oral fungal lesions like denture stomatitis, and numerous candida lesions [27,28]. Chitosan's anti-fungal action is thought to be fungistatic rather than fungicide because it closely mimics the aforementioned antibacterial process. According to research by Ataie et al., nystatin is less efficient than low-molecular-weight chitosan solution for reducing denture stomatitis after two weeks of administration [29]. Chitosan is a promising material based on the positive results of these investigations, the excellent biocompatibility and biodegradability that have been demonstrated, and most crucially the low tendency to create species that are resistant to drugs.

Modification of Oral Hygiene

**Figure 2:**
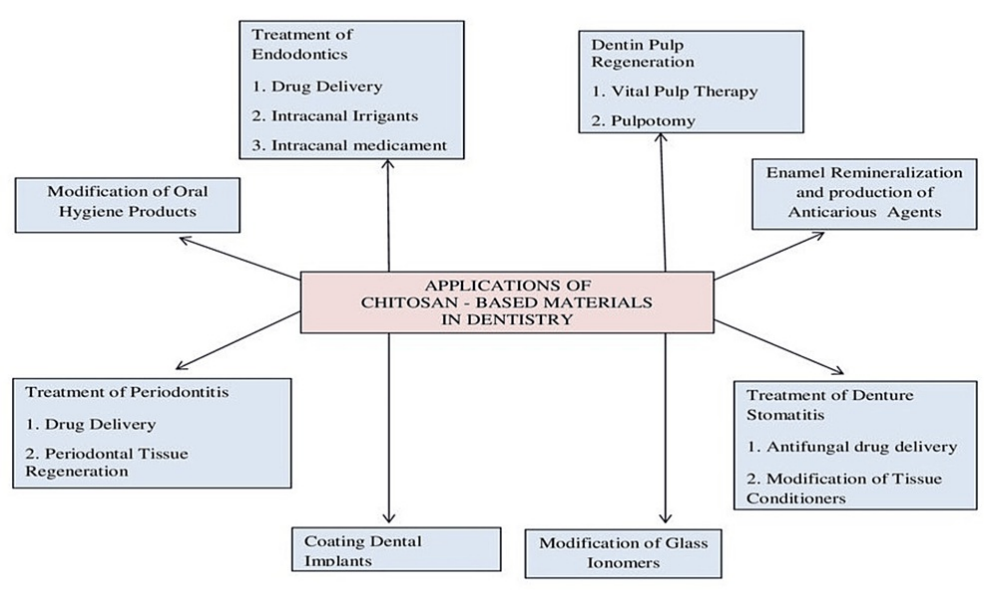
Applications of Chitosan-Based Materials In Dentistry

Dentifrices, mouthwashes, toothpaste, and numerous dental products come in different forms and contain active ingredients that serve multiple functions to enhance oral hygiene and maintain good oral health. In recent years, there has been a growing trend in the oral care industry towards incorporating natural ingredients known for their anti-plaque and anti-caries properties. Costa et al. emphasized the impact of chitosan mouthwash on the attachment of microbes and the development of biofilms involving Prevotella intermedia, Candida albicans, S. mutants, and E. faecalis. This suggests that this mouthwash may be recommended to prevent dental caries, candidiasis, and periodontitis [30]. A new wave of anti-cariogenic formulations, which involve the incorporation of metallic nanoparticles like copper or silver into chitosan, has been a recent subject of investigation [31]. Figure 2 describes the application of CSNPs In different branches of Dentistry.

Limitations of chitosan

Chitosan exhibits limited solubility under alkaline and neutral pH conditions. Its most robust mucoadhesive, as well as permeation-enhancing characteristics, are observed in the duodenal region, and these characteristics can be adjusted through the use of chitosan derivatives. The toxicological characteristics of chitosan derivatives are still being explored. Chitosan has exhibited minimal toxicity to no toxicity in animal studies, and no accounts of significant unfavorable effects in healthy human volunteers. Despite chitosan's approval for use in dietary supplements, wound dressings, and cartilage formulations, there is not a chitosan-based drug formulation approved for widespread commercial distribution at the time of this writing. Challenges related to scaling up production methods will likely draw insights from the development of other polymeric formulations [32]. A summary of all the articles included in this review is listed in Table 1.

**Table 1:** Summary of the articles included in the review

Author	Year	Findings
Mehrabani et al. [1]	2018	Biomaterials have achieved significant progress over recent years.
Zafar et al. [2]	2019	Applications of nanobiomaterials, recent innovations, and potential future expectations are addressed.
Komi and Hamblin [3]	2016	Biosynthesis, nanotechnology and isolation applications are discussed.
Jia et al. [4]	2001	A variety of chitosan derivatives have recently been created to enhance its antimicrobial, antifungal, and antibacterial properties.
Vinsova and Vavrikova [5]	2011	Discusses recent findings on the various activities of chitosan derivatives.
Fakhri et al. [6]	2020	Discusses the role of chitosan in bio-dental materials production and accentuates its current profitable utilizations.
Younes and Rinaudo [7]	2015	The recovery of chitin from marine organisms is described.
Schnurch and Dunnhaupt [8]	2012	Drug delivery systems of chitosan are addressed.
Goosen [9]	2020	Discovery and applications of chitosan are discussed.
Husain et al. [10]	2017	The Application of chitosan has been discussed for the modification and improvement of existing dental materials.
Chandrasekaran et al. [11]	2020	The word "chitosan" typically refers to cationic copolymers.
Ng et al. [12]	2002	The primary product of chitin deacetylation is chitosan.
Varma et al. [13]	2004	Ion binding abilities of Chitosan are addressed.
Sanap et al. [14]	2020	Application of chitosan.
Tavaria et al. [15]	2013	Chitosan as a biomaterial.
Rozman et al. [16]	2019	Antimicrobial activity of chitosan .
Belmonte et al. [17]	1999	Bioactivity of chitosan in dentistry.
Dragland and Kopperud [18]	2015	Antibacterial activity of Chitosan.
ElShiha et al. [19]	2012	Efficacy of chitosan in wound healing after extraction of tooth.
Ardakani et al. [20]	2011	Effects of Chitosan in Bone Repair.
Wieckiewicz et al. [21]	2017	Discusses the applicability and biochemical impact on oral health maintenance.
Park et al. [22]	2000	Chitosan sponge to enhance periodontal bone regeneration.
Erpacal et al. [23]	2019	Bone tissue regeneration will occur more quickly in chitosan-filled dental sockets than in untreated dental sockets.
Park et al. [24]	2003	The beneficial effect of the chitosan/cs on the one-wall intrabony defects.
Kmiec et al. [25]	2017	Discusses the properties of chitosan which could give good results in periodontal care or wound healing.
Malinowska et al. [26]	2017	Application of chitosan in endodontic treatment.
Rodriguez et al. [27]	2019	Antimicrobial activity of endodontic sealers containing chitosan.
Saeed et al. [28]	2019	Combination of chitosan and tissue conditioners is a promising alternative for prevention and treatment of denture stomatitis.
Atai et al. [29]	2017	Antifungal effect of Chitosan against Candida Albicans.
Costa et al. [30]	2014	Impact of Chitosan Mouthwash on oral microorganisms.
Giulio et al. [31]	2013	The Effect on Streptococcal and Saliva-Derived Biofilms by silver nanoparticle polysaccharide system.
Chaudhury and Das [32]	2011	Limitations and recent advancements in the chitosan.

## Conclusions

Chitosan, a biopolymer with distinctive attributes and a diverse array of applications in biomedicine, along with its derivatives, has been the focus of extensive research. Nevertheless, several unresolved issues remain, warranting further research and exploration. Chitosan stands as an innovative biomaterial with a broad range of applications in dentistry, encompassing everything from restorative procedures to the creation of tissue-engineered scaffolds for alveolar bone, as well as aiding in the healing of the periodontal complex. Nonetheless, this constantly advancing field of dentistry can harness the potential of this derived polymer in various other areas, including orthodontic, prosthetic, and implant-related applications. Hence, there is a significant capability for broadening its biological utilization in the future. However, there is a scarcity of clinical data available related to the practical dental role of chitosan-based materials. To transition chitosan-based materials from research to clinical roles, additional investigation is imperative, especially in the realms of in vivo studies and clinical trials. Probable future studies may carry out the role of chitosan in practical applications in dentistry, and advances in chitosan restorative materials which are necessary for its success in dentistry. Research in the field of CSNPs can be very advantageous in the future growth of new techniques, and applications in dentistry. However, there is a need for investigation concerning safety issues and toxicity of CSNPs which should be addressed in future studies. Chitosan has been the focus of extensive study owing to its exceptional properties as a biopolymer. Over the years, it has spurred new research endeavors in the field of tissue engineering. It has been demonstrated that chitosan possesses sufficient ability to serve as a substitute for skin, as well as in antimicrobial, blood anticoagulation, regeneration, anti-inflammatory, and bone healing, among numerous other fantastic applications.
